# 1α,25(OH)_2_D_3_-glycosides from *Solanum glaucophyllum* leaves extract induce myoblasts differentiation through p38 MAPK and AKT activation

**DOI:** 10.1242/bio.033670

**Published:** 2018-04-23

**Authors:** Ana Paula Irazoqui, Pablo De Genaro, Claudia Buitrago, Heinrich Bachmann, Verónica González-Pardo, Ana Russo de Boland

**Affiliations:** 1 Instituto de Ciencias Biológicas y Biomédicas del Sur (INBIOSUR), Universidad Nacional del Sur-CONICET, 8000 Bahía Blanca, Argentina; 2 Departamento de Biología, Bioquímica y Farmacia, Universidad Nacional del Sur, 8000 Bahía Blanca, Argentina; 3 Herbonis AG, Rheinstrasse 30, CH-4302 Augst, Switzerland

**Keywords:** C2C12 cells, 1α,25(OH)2D3-glycosides, P38 MAPK, AKT, MyoD1, Myogenin, MHC2b, Proliferation

## Abstract

We have previously shown that *Solanum glaucophyllum* leaf extract (SGE) increases VDR protein levels and promotes myoblast differentiation. Here, we investigated whether p38 MAPK and AKT are involved in SGE actions. Cell-cycle studies showed that SGE prompted a peak of S-phase followed by an arrest in the G0/G1-phase through p38 MAPK. Time course studies showed that p38 MAPK and AKT phosphorylation were statistically increased by SGE (10 nM) or synthetic 1α,25(OH)_2_D_3_ (1 nM) treatment. Furthermore, p38 MAPK and AKT inhibitors, SB203580 and LY294002 respectively, suppressed myoblasts fusion induced by SGE or synthetic 1α,25(OH)_2_D_3_. We have also studied differentiation genes by qRT-PCR. *myoD1* mRNA increased significantly by SGE (24–72 h) or 1α,25(OH)_2_D_3_ (24 h) treatment. mRNA expression of *myogenin* also increased upon SGE or 1α,25(OH)_2_D_3_ treatment. Finally, *MHC2b* mRNA expression, a late differentiation marker, was increased significantly by both compounds at 72 h compared to control. Taken together, these results suggest that SGE, as synthetic 1α,25(OH)_2_D_3_, promotes myotube formation through p38 MAPK and AKT activation.

## Abstract (precis)

**Summary:** *Solanum glaucophyllum* leaf extract (SGE) contains 1α,25-dihydroxyvitamin D_3_-glycosides that promotes myoblast fusion through AKT and p38 MAPK pathways. Moreover, SGE regulates the expression of myoD1, myogenin and MCH2b at the beginning of differentiation.

## INTRODUCTION

The biologically active form of vitamin D is 1α,25-dihydroxyvitamin D_3_ [1α,25(OH)_2_D_3_], and it plays a key role in calcium and phosphorus metabolism. More recently, important pleiotropic effects have been described including: cell proliferation inhibition, differentiation, anticarcinogenic effects, innate-stimulation and adaptive immunity inhibition, inflammation inhibition as well as several other endocrine and developmental effects (Hewison, 2012; Plum and DeLuca, 2010; Bikle, 2011; Hii and Ferrante, 2016). This form binds to the vitamin D receptor (VDR) and regulates gene expression in a ligand-dependent manner (Haussler et al., 2011; Hii and Ferrante, 2016). VDR is expressed in skeletal muscle tissue at different stages, from myoblasts (mononucleated cells) to myotubes (multinucleated cells). Furthermore, the evidence of VDR presence has been reported in rodent skeletal muscle cell lines (Simpson et al., 1985), in chicken myoblasts monolayers (Boland et al., 1985) and in human skeletal muscle cells (Costa et al., 1986).

The traditional activity of 1α,25(OH)_2_D_3_ to modulate gene transcription is also complemented with its ability to regulate intracellular, extranuclear pathways and cytoplasmic signaling cascades. These cytoplasmic actions are generally regarded to be rapid responses, taking place from within seconds to minutes. Numerous studies detail the ability of 1α,25(OH)_2_D_3_ to affect intracellular calcium levels as well as intracellular signaling pathways involving kinases and phosphatases. The activation of different signaling pathways is dependent on the cell type, thus providing an avenue to explain the pleiotropic effects of vitamin D (Wang and Sun, 2009; Audo et al., 2003; Sidelnikov et al., 2010; Hii and Ferrante, 2016). Low vitamin D levels are associated with different degrees of muscle dysfunction; consequently, supplementation with vitamin D has generally shown beneficial effects. Among dietary supplements the use of vitamin D_3_ has been shown to improve bone health and muscle strength of broilers (Papesova et al., 2008; Michalczuk et al., 2010; Garcia et al., 2013), feedlot steers’ health (Montgomery et al., 2004) and meat tenderness (Montgomery et al., 2002). However, 1α,25(OH)_2_D_3_ is not available for animal nutrition because of high costs, but it has been found that a few plants species have 1α,25(OH)_2_D_3_ and other vitamin D_3_ metabolites (Boland et al., 2003). *Solanum glaucophyllum* plants accumulate 1α,25(OH)_2_D_3_-glycosides, and so their leaves become a natural source of the active form of vitamin D. *S.* *glaucophyllum* leaf extract (SGE) is used in a cold water solution for poultry breeding (Bachmann et al., 2013). One advantage of SGE is that 1α,25(OH)_2_D_3_ can become available quickly when entering the body as there is no need for the two metabolic steps to become active (Rambeck et al., 1981). The active component present in the plant extract has been shown to induce calcium and phosphorus transport and thus it is recommended for treatment of liver and/or kidney function impairment in older animals and stress conditions (Bachmann et al., 2013; Edwards, 1989, 1993).

We have previously demonstrated that SGE increases VDR, myogenin, and myosin heavy chain protein levels during the first 48 h of C2C12 muscle cell line differentiation (Gili et al., 2016). Moreover, SGE improves the growth and differentiation of C2C12 cells at the onset of myogenesis, increasing cellular mass and myotube fusion (Gili et al., 2016). In this work, we further studied the effects of SGE on the signaling pathways involved in C2C12 muscle cell differentiation.

## RESULTS AND DISCUSSION

**Fig. 1.**
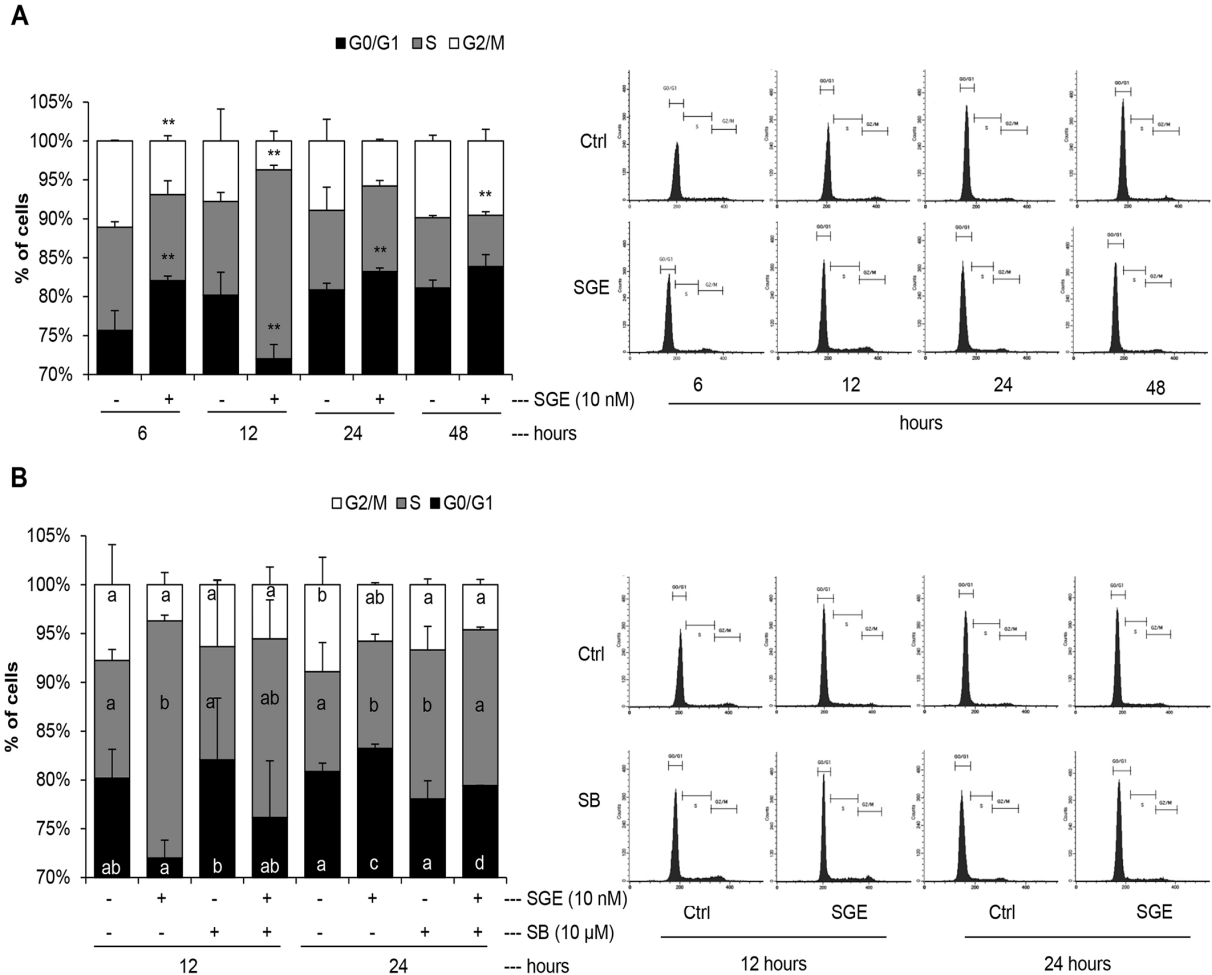
**Cell cycle progression of C2C12 skeletal muscle cells after SGE treatment: role of p38 MAPK.** A. This panel shows a representative histogram and DNA quantification from three independent experiments showing the percentage of cells in G0/G1-, S- and G2/M-phases (Y-axis label) ±s.d. Data were analyzed by one way ANOVA, followed by *t*-test, **P<0.01. B. This panel presents a representative histogram and DNA quantification from four independent experiments expressing the percentage of cells in G0/G1-, S- and G2/M-phases (Y-axis label) ±s.d. in combined bars graphs. Different letters mean statistical differences with Tukey test.

Skeletal muscle differentiation involves a complex process where a broad number of signal pathways are coordinated. At the beginning of the differentiation, satellite cells (muscle stem cells) commit to myogenic precursor cells known as myoblasts. Successively, a series of regulatory factors collaborate to differentiate the myoblasts. To complete this process, mononuclear cells or myocytes align and fuse to form multinuclear cells or myotubes (Ge et al., 2013; Miyake et al., 2011). To further investigate the mechanism of SGE on the differentiation of muscle cells, the murine skeletal muscle cell line C2C12 was chosen. The regulation of the cell cycle in C2C12 muscle cells treated with fetal bovine serum (FBS) or synthetic 1α,25(OH)_2_D_3_ has been previously reported (Irazoqui et al., 2014). In the present study, we first examined the effect of SGE on C2C12 cell cycle progression. To that end, cells were deprived of FBS for 16 h to synchronize the cultures at a time in which 85% of the cells were growth-arrested in the G0/G1-phase, as it was previously described (Irazoqui et al., 2014). Then, arrested cells were stimulated with 10 nM of SGE for different periods of time (6–48 h) and analyzed by flow cytometry. Fig. 1A shows that the percentage of cells in the G0/G1-phase increased after 6 h of SGE treatment whereas the G2/M phase declined. Progression of the cell cycle from G1 to S-phase continued and an S-phase peak was observed at 12 h. After completion of the S-phase, an increase in the G0/G1-phase was observed at 24 h, showing that one round of the cell cycle had been completed and cells had begun preparing to start posterior differentiation. This cell cycle pattern prompted by SGE was similar to the one reported in C2C12 stimulated with synthetic 1α,25(OH)_2_D_3_ (Irazoqui et al., 2014).

Several downstream signaling pathways, including p38 MAPK, Rho family small GTPases and AKT, are implicated in cell myogenesis (Gonzalez et al., 2004; Takaesu et al., 2006; Bryan et al., 2005). Transient or sustained MAPKs activation regulates cellular fate mechanisms such as proliferation and differentiation. The best characterized mammalian MAPKs are grouped into four main modules, the ERK1/ERK2, JNK, p38 and ERK5 modules, but more subgroups might exist (Kyriakis and Avruch, 2012). When activated, MAPKs translocate into the nucleus and induce transcription factor phosphorylation either directly or indirectly through downstream kinases (Kyriakis and Avruch, 2012). p38 MAPK has been identified as an important signaling pathway to promote skeletal muscle differentiation. Furthermore, p38 MAPK inhibition abrogates myoblast fusion and differentiation (Segalés et al., 2016). Based on this observation, we next investigated whether p38 MAPK takes part in the S-phase peak and later G0/G1 arrest promoted by SGE in C2C12 cells. To achieve this, first C2C12 cells were cultured in growth medium (GM), and then this medium was changed to differentiation medium (DM) to induce differentiation. Cells were treated with synthetic SGE (10 nM) in the presence or absence of p38 MAPK inhibitor SB 203580 (10 µM) or vehicle alone for 12 h and 24 h. As illustrated in Fig. 1B, SGE-induced G0/G1 cell arrest was abrogated when p38 MAPK was previously inhibited, showing that SGE activates the p38 MAPK pathway to promote the onset of muscle differentiation.

**Fig. 2.**
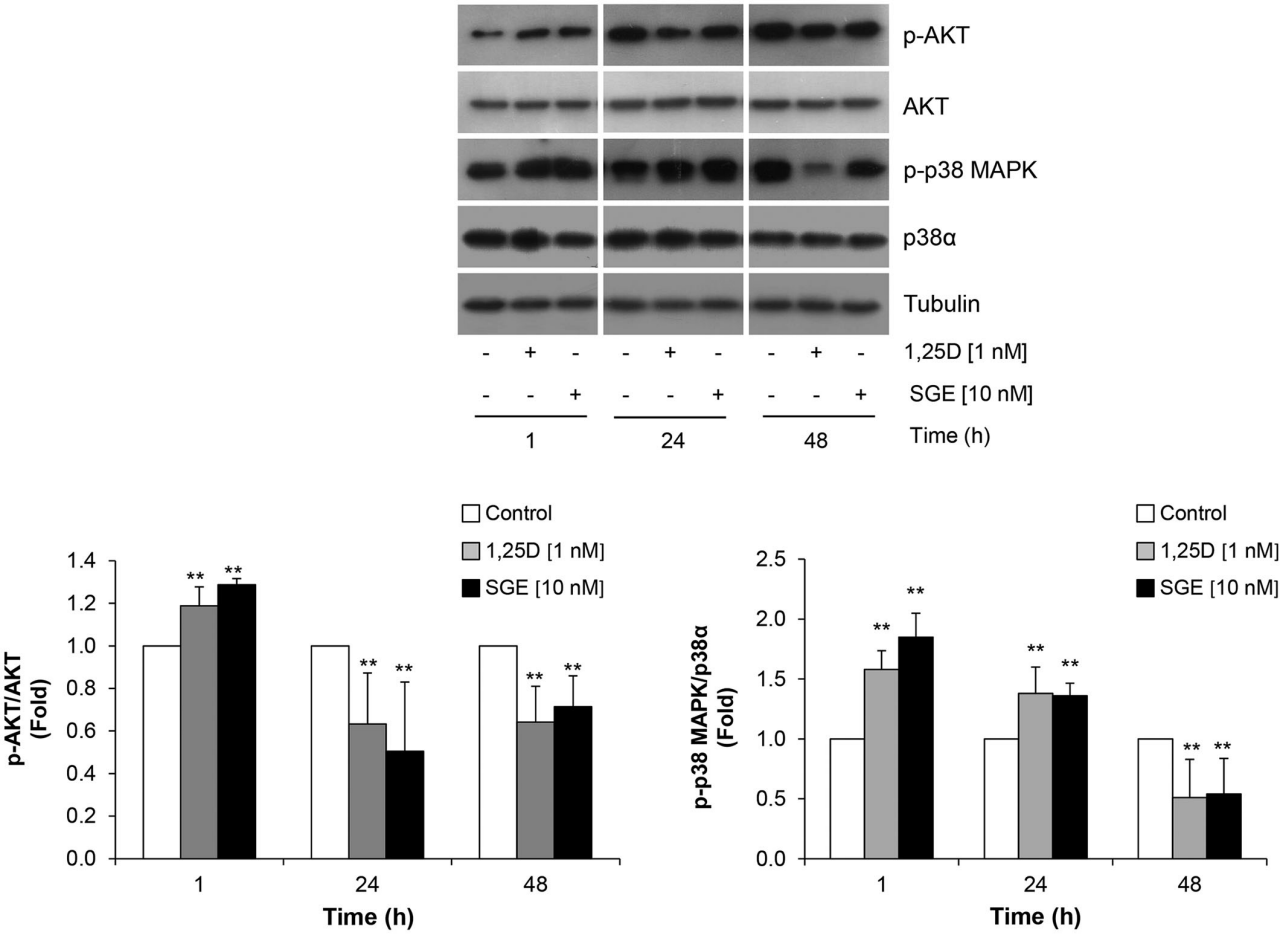
**SGE regulates AKT and p38 MAPK phosphorylation at the beginning of myogenesis.** C2C12 cells were cultured in GM. To begin the differentiation, this medium was withdrawn and then replaced by DM. Treatment was started by adding 10 nM of SGE or 1 nM of synthetic 1α,25(|OH)_2_D_3_ (1,25D) in DM during different periods of time (1–48 h). Western blots were performed with anti p-AKT, total AKT, p-p38 MAPK, and p38α and tubulin antibodies. Tubulin was used as loading protein marker. Representative blots from three independent experiments (upper panel) and their quantification (lower panel) are shown. Protein bands quantification was done using ImageJ. Results were then represented in bar graphs as phosphorylated protein normalized with its corresponding total from treated conditions and referred to control (Fold) (lower panel). Significant differences between conditions at each time point were analyzed by Student’s *t*-test, **P<0.01.

It has been reported that 1α,25(OH)_2_D_3_ stimulates the PI3K-Akt pathway (Dwivedi et al., 2010) and other well-supported evidence has suggested that AKT signaling has an important role in myoblast differentiation (Gonzalez et al., 2004; Fujio et al., 2001; Vandromme et al., 2001). It was reported that AKT overexpression enhances myoblast differentiation, whereas AKT inhibition, by expression of a dominant-negative AKT, blocks myotube formation. The suppression of myogenesis caused by PI3-kinase inhibition is rescued by the ectopic expression of a constitutively active AKT (Tureckova et al., 2001)*.* AKT participation in C2C12 differentiation by 1α,25(OH)_2_D_3_ was previously reported in C2C12 (Buitrago et al., 2012). However, an experimental approach different from ours was used. In that report, cells were grown in DM medium for 48 h before 1α,25(OH)_2_D_3_ (1 nM) treatment, and thus a different pattern of AKT phosphorylation was observed. In view of p38 MAPK and AKT implication in muscle cells differentiation, we then studied whether SGE regulates p38 MAPK and AKT likewise regulates 1α,25(OH)_2_D_3_. To that end, time course experiments were performed during SGE (10 nM) or synthetic 1α,25(OH)_2_D_3_ (1 nM) treatment at the beginning of C2C12 differentiation (1–48 h). Proteins from whole cell lysates were subjected to SDS-PAGE and p38-MAPK, and AKT protein phosphorylation was analyzed by Western blot. The results presented in Fig. 2 show that AKT phosphorylation increased rapidly at 1 h and then decreased significantly after SGE or 1α,25(OH)_2_D_3_ treatment (24–48 h), whereas p38 MAPK phosphorylation also increased at 1 h and remained elevated for 24 h to decrease later (48 h). Our study supports the hypothesis that the effects of SGE in AKT phosphorylation are similar to synthetic 1α,25(OH)_2_D_3_.

Myoblast fusion is essential for the development of skeletal muscle myofibers. The formation of multinucleated muscle cells through cell-fusion is a conserved mechanism from fruit flies to humans. Cell-cell fusion of myoblasts give rise to the functional unit of muscle, the multinucleated myofiber (Aguilar et al., 2013).

We have previously shown that SGE as synthetic 1α,25(OH)_2_D_3_ induces myoblasts fusion into myotubes (Gili el at., 2016)*.* Results from our laboratory have indicated that p38 MAPK activation by 1α,25(OH)_2_D_3_ is VDR-dependent in C2C12 myoblasts under a proliferative stage (Buitrago et al., 2013). It was also shown that c-Src-dependent stimulation of MKK3 and 6 rapidly activates p38 MAPK, which in turn phosphorylates MAPK2 that subsequently promotes the phosphorylation of heat-shock protein 27 in C2C12 myoblasts. In agreement with our findings, p38 MAPK involvement in cell-cycle control of C2C12 cells stimulated with 1 nM of 1α,25(OH)_2_D_3_ has been previously demonstrated, providing key information of a 1α,25(OH)_2_D_3_ mechanism in myogenesis regulation (Buitrago et al., 2013). Thus, our study demonstrates SGE’s effect on signaling pathways involved in myogenesis.

**Fig. 3.**
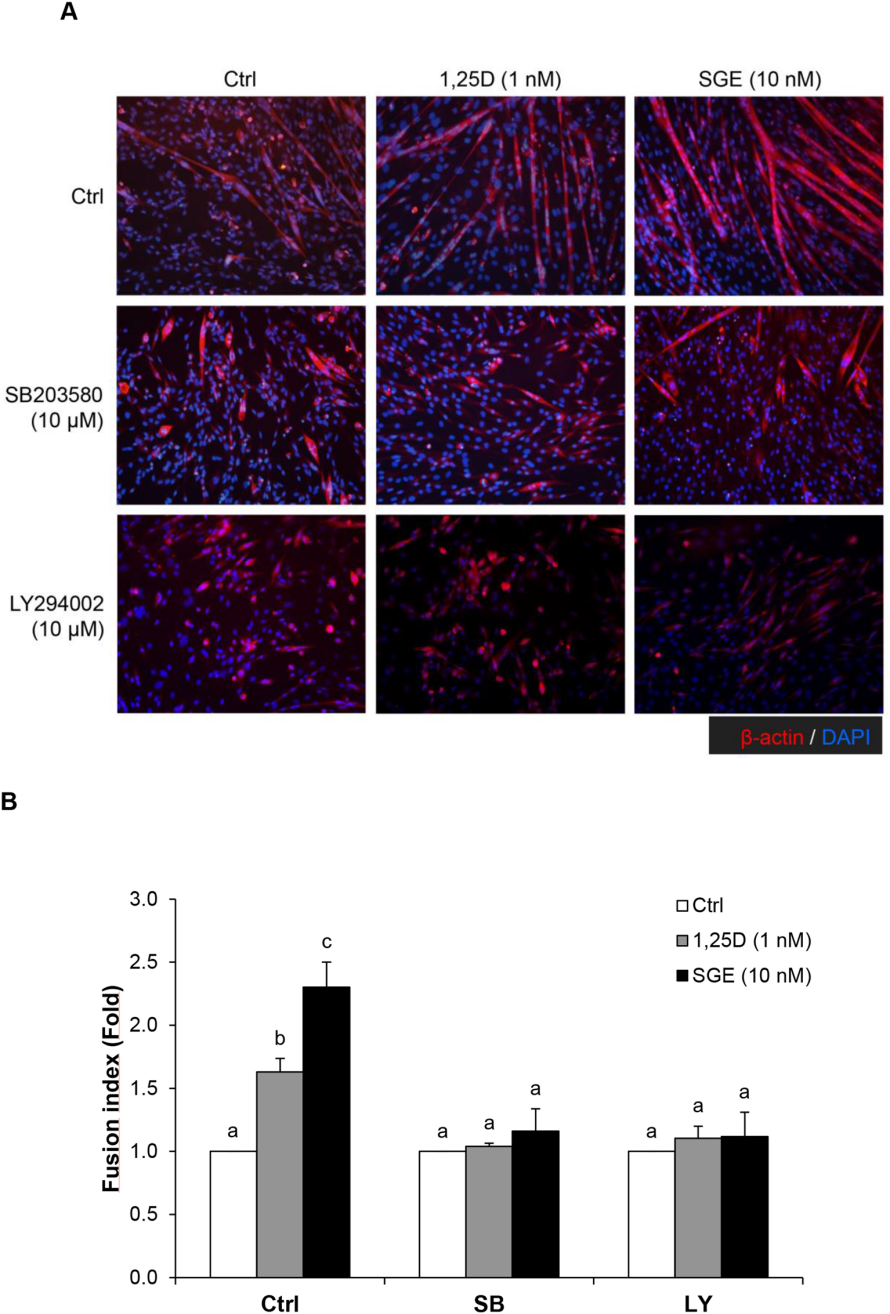
**SB203580 and LY294002 inhibitors prevent** **myoblast** **differentiation induced by SGE or synthetic 1α,25(OH)_2_D_3_.** (A) Fluorescence photomicrographs show C2C12 cells undergoing differentiation for 72 h. Cells were treated with vehicle (Ctrl, control), 1 nM of 1α,25(OH)_2_D_3_ (1,25D), or 10 nM of SGE, or in presence of SB203580 (10 µM), or LY294002 (10 µM), as described in the text. (B) Fusion index was calculated and represented in bar graphs from three independent experiments. Significant differences between conditions were analyzed by one way ANOVA followed by Bonferroni Test. Different letters indicate statistical differences among groups for each condition (*P*<0.05).

Four isoforms of p38 MAPK have been described; Wang and collaborators have found that p38α, β and γ are required for C2C12 cell differentiation (Wang et al., 2008). SB203580 specifically inhibits p38 isoforms α and β and has been successfully used to elucidate the role of these isoforms. Results obtained using this inhibitor have implicated p38α and β in skeletal muscle differentiation *in vitro* (Kumar et al., 1997). p38 MAPK signal enables myoblasts to exit the cell cycle at the beginning of differentiation (Lee et al., 2002). Since p38α is a key signaling pathway involved in skeletal muscle differentiation and inhibition of p38α activity abrogates myoblast differentiation and fusion (Segalés et al., 2016), we assessed whether p38 MAPK, activated by SGE, takes part in the fusion of C2C12 cells to form myotubes during differentiation. To that end, C2C12 cells were induced to differentiate in DM and treated with SGE (10 nM) or synthetic 1α,25(OH)_2_D_3_ (1 nM) in the presence or absence of SB203580 (10 µM), or vehicle alone for 72 h. At the end of the treatment, cells were fixed and incubated with anti-β-actin antibody and DAPI, and then examined using a fluorescence microscope. Images were collected and the fusion index was calculated as described in the Materials and Methods. As illustrated by Fig. 3, myotubes were developed in cells exposed to DM for 72 h (control) and it was statistically and similarly increased by either SGE or 1α,25(OH)_2_D_3_. When cells were incubated with SB203580, cell fusion was blocked. Moreover, when SB203580 was used in combination with SGE (10 nM) or 1α,25(OH)_2_D_3_ (1 nM), the ability of both compounds to induce myotube formation was impaired (Fig. 3B). As reported by Irazoqui et al. (2014), our studies confirm that p38α and β treated by 1α,25(OH)_2_D_3_ are implicated in the withdrawal of myoblasts from the cell cycle to promote differentiation. Furthermore, our results expand the knowledge of the SGE mechanism that triggers myoblast fusion through p38 MAPK.

Since AKT activation induced by synthetic 1α,25(OH)_2_D_3_ in skeletal muscle differentiation was previously reported (Buitrago et al., 2012), to study AKT participation in myotube fusion promoted by SGE, the inhibitor LY294002 was used. LY294002 (10 µM) has been shown to effectively block PI3K activation upstream of AKT in C2C12 cells by other authors (Gamell et al., 2008). As indicated in Fig. 3A and B, inhibition of AKT activity blocked myotube fusion induced by SGE as well as 1α,25(OH)_2_D_3_, showing that AKT signaling is also part of SGE mechanism to trigger C2C12 differentiation.

**Fig. 4.**
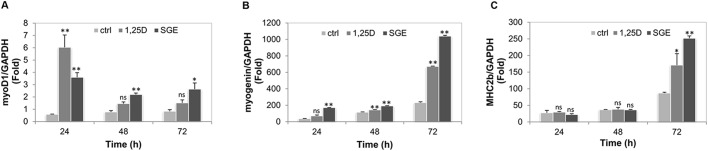
**Genes regulation by SGE or synthetic 1α,25(OH)_2_D_3_ during onset of differentiation.** C2C12 cells were treated with SGE (10 nM), 1α,25(OH)_2_D_3_ (1,25D, 1 nM), or vehicle alone (ctrl) in DM in time course experiments (24–72 h). Total RNA was extracted and reverse transcribed and data analysis was performed for each gene expression by qRT-PCR. Bar graphs show quantitative results from three experiments performed in duplicate and expressed as a ratio between mRNA of each gene (A) *myoD1*, (B) *myogenin*, (C) *MHC2b* in DM under control conditions (ctrl) or treated (1,25D or SGE) previously normalized to GAPDH mRNA levels and to GM (time 0). The results were then represented in bar graphs. Significant differences between control and treated (SGE or 1,25D) conditions at each time point were analyzed by Student's *t*-test. ns: not significant. *P<0.05 and **P<0.01.

Until now, myogenic regulation is a theme of study. The precise molecular mechanism is being explored and many novel genes and factors are emerging as new candidates. In this regard, a number of transcription factors and muscle-specific genes have been confirmed as muscle determination factors (Brown et al., 2012; Wang et al., 2012a, b). In this context, we next studied the pattern of *myoD1*, *myogenin* and *MCH2b* gene expression during the differentiation phase. C2C12 were cultured for 24 h in GM, which was then replaced with DM and SGE (10 nM), 1α,25(OH)_2_D_3_ (1 nM) or vehicle was added for 24–72 h. mRNA gene expression was then analyzed by qRT-PCR. As can be observed in Fig. 4A, *myoD1* mRNA control levels remained low throughout the period of analysis whereas in treated conditions, *myoD1* mRNA increased upon the addition of SGE or 1α,25(OH)_2_D_3_. SGE-induced mRNA synthesis was sustained during the period of analysis in contrast to synthetic 1α,25(OH)_2_D_3_ that only increased significantly at 24 h (Fig. 4A). The early differentiation marker *myogenin* also increased upon SGE or 1α,25(OH)_2_D_3_ treatment, however, *myogenin* mRNA induction by 1α,25(OH)_2_D_3_ started at 48 h in contrast to SGE (Fig. 4B). Finally, the late differentiation marker, *MHC2b*, also increased significantly by SGE or 1α,25(OH)_2_D_3_ at 72 h compared to control (Fig. 4C).

In conclusion, our results indicate that SGE regulates the cell cycle and promotes myotube fusion through p38 MAPK and AKT regulation in skeletal muscle C2C12 cells. Moreover, both vitamin D metabolites, SGE and synthetic 1α,25(OH)_2_D_3_, modulate gene expression during myogenesis.

## MATERIALS AND METHODS

### Chemicals and reagents

We obtained 1α,25(OH)_2_D_3_ from Cerbios AG, Lugano, Switzerland (http://www.cerbios.ch) and an extract of dry leaves of the plant *S.* *glaucophyllum*, containing 50 µg g^−1^ analytically determined 1α,25(OH)_2_D_3_, was kindly provided by Herbonis AG (Augst, Switzerland). Dulbecco's modified Eagle's medium (DMEM) low glucose, with l-glutamine and HEPES, without phenol red, Immobilon P (PVDF) membranes, LY294002 and SB 203580 were from Sigma-Aldrich. Sterile fetal bovine serum (FBS) and horse serum (HS) were purchased from Natocor (Córdoba, Argentina). The antibodies used were: anti-p-p38 MAPK (4511), p-AKT (9271), AKT (4685) from Cell Signaling Technology; Goat anti-Rabbit, IgG-HRP (sc-2004) and anti-β-actin (sc-47778) antibodies from Santa Cruz Biotechnology. DAPI, β-tubulin antibody, Goat anti-Mouse IgG (H+L) Cross-Adsorbed Secondary Antibody, Cyanine3 were from Thermo Fisher Scientific. PCR primers, Superscript III reverse transcriptase (Invitrogen), Sybr green PCR master mix (Applied Biosystems) were also from Thermo Fisher Scientific. High Pure RNA Isolation Kit was from Roche (F. Hoffmann-La Roche Ltd., Madison, USA).

### Cell culture and treatment

We used the murine skeletal muscle cell line C2C12 to study myogenesis (Gili et al., 2016). Cells were cultured with DMEM supplemented with 10% heat-inactivated (30 min, 56°C) FBS, 1% nystatine, and 1% streptomycin, GM at 37°C under a humidified atmosphere of 5% CO_2_ in air. C2C12 myoblasts cultured in these conditions resemble the activated satellite cells (Yoshida et al., 1998). Myoblast differentiation was promoted as was previously reported (Gili et al., 2016). Briefly, (with C2C12 cells at 70–80% of confluence) GM was replaced with DMEM without phenol red, supplemented with 2% HS (DM). C2C12 cells were incubated in DM with 1α,25(OH)_2_D_3_ (1 nM) or SGE (10 nM) dissolved in less than 0.01% ethanol as vehicle during the experimental period.

### Cell lysates and protein content determination

C2C12 cells were scraped in 75 µl of lysis buffer and lysates were collected by aspiration and centrifuged at 14,000 ***g*** for 15 min (Gili et al., 2016). Proteins from supernatant were quantified using the Bradford procedure as before (Gili et al., 2016; Bradford, 1976).

### Western blot analysis

Proteins were separated on SDS-PAGE (10% gels) and electro transferred to PVDF membranes. Membranes were then blocked and subjected to immunoblotting (Gili et al., 2016). Incubation with primary antibodies, (1:1000) in 0.1% TBST containing 2.5% dry milk, was performed overnight at 4°C. After washing three times with 0.1% TBST, membranes were incubated with secondary antibodies conjugated with peroxidase (1:5000) in TBST buffer as primary antibodies, for 1 h at room temperature. Blot signals were visualized using an enhanced chemiluminescence technique (ECL) according to the manufacturer's instructions (Amersham ECL Western Blotting Detection Kit, GE Healthcare). Images were scanned and quantified using ImageJ software.

### Fluorescence microscopy

Cells were fixed with formaldehyde for 15 min. Fixed cells were then incubated with anti-β-actin (1:50) overnight at 4°C and then with Goat anti-Mouse IgG (H+L) Cross-Adsorbed Secondary Antibody, Cyanine3 for 1 h and then with DAPI (1:1000) of a stock solution of 5 mg ml^−1^ for 30 min at room temperature, in darkness. Samples were then washed three times with phosphate-buffered saline (PBS), mounted and examined using NIKON Eclipse Ti-S fluorescence microscope equipped with standard filter sets (Gili et al., 2016). Images were collected using a digital camera.

### Fusion index determination

Images collected from Fluorescence microscopy were merged with Fiji software (ImageJ) and the fusion index was calculated as in our previous work (Gili et al., 2016). Briefly, the fusion index (number of nuclei in myotubes/total number of nuclei) was calculated by counting nuclei in ten random fields from three culture dishes (five fields in each dish).

### Cell cycle analysis by flow cytometry

C2C12 cells were collected by trypsinization, washed with PBS twice and then fixed in 70% of ethanol. Fixed cells were washed in PBS and re-suspended with 500 µl of RNase-propidium iodide mix (BD Biosciences) for 30 min. The fluorescence of DNA was measured using a flow cytometer (BD FACSCalibur) and the cell distribution in different phases of the cellular cycle was analyzed by computer software (CELLQuest PRO, Becton Dickinson, San Jose, USA).

### RNA Isolation and quantitative RT-PCR

Total RNA for quantitative RT-PCR (qRT-PCR) analysis was isolated with the High Pure RNA Isolation Kit (Roche) following the procedure provided by manufacturer. Then 1 µg of RNA was reverse transcribed using the Superscript III Reverse transcriptase and qRT-PCR reactions were performed on the resulting cDNA in a 7500 Fast Real Time PCR system (Applied Biosystems). Specific primers were used to detect *myoD1*, *myogenin* and *MHC2b* mRNA levels. *Gapdh* mRNA was used to normalize gene expression. Data of relative fold change was obtained by the comparative CT method. Oligonucleotides used for amplification were: murine *myoD1*: forward 5′-CAACGCCATCCGCTACATCG-3′ and reverse 5′-GTCCAGGTGCGTAGAAGGCA-3′; murine *myogenin*: forward 5′-TGCCGTGGGCATGTAAGGT-3′ and reverse 5′-TGCGCAGGATCTCCACTTTAG-3′ and murine *MHC2b*: forward 5′-CAATCAGGAACCTTCGGAACAC-3′ reverse 5′-CAATCAGGAACCTTCGGAACAC-3′ and murine *Gapdh*, forward 5′-GAAGGTGAAGGTCGGAGTC-3′ and reverse 5′-GAAGATGGTGATGGGATTTC-3′.

### Statistical analysis

Data are shown as mean±s.d. Cell cycle statistical analysis was performed by one way ANOVA, followed by Tukey test. Index fusion was analyzed by one way ANOVA, followed by Bonferroni test. Different superscript letters indicate significant differences at *P*<0.05. Data from DNA quantification in Fig. 1A, time-response studies obtained by qRT-PCR or Western blot were analyzed by the two-tailed *t*-test. **P< 0.01 and *P< 0.05 were considered statistically significant.
